# Polypharmacy and Mental Health Issues in the Senior Hemodialysis Patient

**DOI:** 10.3389/fpsyt.2022.882860

**Published:** 2022-05-12

**Authors:** Maša Knehtl, Tadej Petreski, Nejc Piko, Robert Ekart, Sebastjan Bevc

**Affiliations:** ^1^Department of Nephrology, University Medical Center Maribor, Maribor, Slovenia; ^2^Faculty of Medicine, University of Maribor, Maribor, Slovenia; ^3^Department of Dialysis, University Medical Center Maribor, Maribor, Slovenia

**Keywords:** chronic kidney failure, seniors, polypharmacy, mental health, hemodialysis, psychotropic medications, pharmacokinetics

## Abstract

Hemodialysis (HD) is the most common method of chronic kidney failure (CKF) treatment, with 65% of European patients with CKF receiving HD in 2018. Regular two to three HD sessions weekly severely lower their quality of life, resulting in a higher incidence of depression and anxiety, which is present in one third to one half of these patients. Additionally, the age of patients receiving HD is increasing with better treatment and care, resulting in more cognitive impairment being uncovered. Lastly, patients with other mental health issues can also develop CKF during their life with need for kidney replacement therapy (KRT). All these conditions need to receive adequate care, which often means prescribing psychotropic medications. Importantly, many of these drugs are eliminated through the kidneys, which results in altered pharmacokinetics when patients receive KRT. This narrative review will focus on common issues and medications of CKF patients, their comorbidities, mental health issues, use of psychotropic medications and their altered pharmacokinetics when used in HD, polypharmacy, and drug interactions, as well as deprescribing algorithms developed for these patients.

## Introduction

Most hemodialysis (HD) patients have other common chronic conditions in addition to chronic kidney failure (CKF), including arterial hypertension (AH), diabetes mellitus (DM), cardiovascular disease (CVD), and mineral and bone disorder (MBD), all of which require long-term pharmacologic management. HD patients take on average 10–12 prescribed and over-the counter medications from an average of 4.7 prescribers, and an average of 19 pills per day (1). They tend to be older, have a high symptom burden, and multiple comorbidities. This often leads to polypharmacy resulting in possible drug-drug interactions (DDI) (2).

The term “polypharmacy” has no single definition but is generally used to refer to the use of four or more regular medications by older adults, which may lead to several Medication-Related Problems (MRPs) or excessive or unnecessary drug therapy. Several categories of MRPs were described in chronic kidney disease (CKD) patients, including: (a) untreated indications, (b) improper drug selection, (c) improper drug dosing, (d) adverse drug reactions, (e) DDI, (f) adherence, and (g) drug use without indication (1, 3). MRPs may increase hospital admissions, morbidity, mortality, and pose a high financial burden to the healthcare system (3).

When dialysis physicians use the term “polypharmacy” regarding the HD patient, they generally mean the second definition of excessive or unnecessary drug therapy. Such excess often leads to unintended consequences, such as additional risks for DDI or drug-food interactions or adverse effects. Adverse effects can lead to further use of unnecessary therapy when additional drugs are added to combat those consequences (1).

Polypharmacy also likely contributes to cognitive impairment. The high absolute number of medications, combined with potential for DDI and impaired kidney clearance, creates a high risk for sedation, delirium, and cognitive impairment (2). A general rule of prescribing medications to the patient with CKF is to start with the lowest dose, use longer dosing intervals and increase the dose slowly while monitoring for efficacy and features of toxicity (4). Drugs cleared by HD should be given after the procedure (4).

Common symptoms of chronic HD patients include pain, uremic pruritus, fatigue, and sleep disturbances (4). Considering drug therapy for pain, the use of systemic non-steroidal anti-inflammatory drugs is generally contraindicated. Paracetamol is the initial analgesic of choice, with no necessary dose modification. Furthermore, paracetamol remains a useful background treatment even when opioids are required. Opioids must be used carefully in renal supportive care, given their narrow therapeutic window and potential for accumulation and toxicity. Gabapentin and pregabalin (gabapentinoids) are the preferred initial therapy for neuropathic pain. Due to their almost exclusive renal elimination, dose reductions are compulsory. Monitoring for the common adverse effects of somnolence, dizziness, and gait disturbance is important. Additionally, tricyclic antidepressants, such as amitriptyline, can be used to manage neuropathic pain. Furthermore, serotonin and norepinephrine reuptake inhibitors (SNRIs) such as duloxetin can also be used (4). Gabapentinoids are also the first-line drug therapy for restless legs syndrome. An extra dose can be taken 1 h before the HD procedure if the patient is symptomatic during HD (4). Gabapentinoids also have the strongest supporting evidence in generalized uremic pruritus. Possible alternatives include sertraline or doxepin (4). Another challenging field is cooperating with the HD patient with cognitive impairment or other mental illness.

**FIGURE 1:**
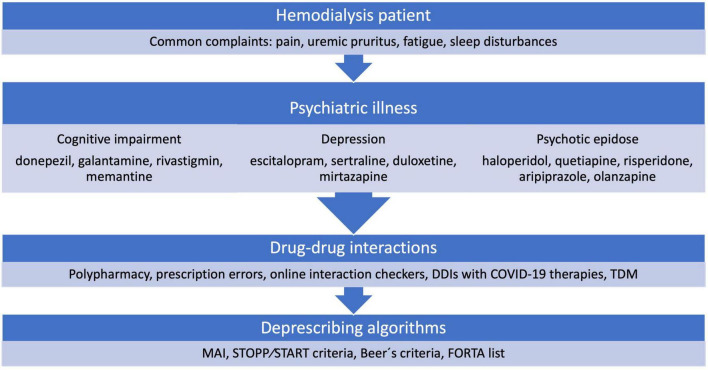
Overview of the covered topics. DDIs, drug-drug interactions; TDM, therapeutic drug monitoring; MAI, Medication Appropriateness Index; STOPP/START, Screening Tool of Older Persons Prescriptions and Screening Tool to Alert doctors to Right Treatment; FORTA, Fit-fOR-The-Aged.

The aim of this review was to investigate the literature that focuses on selected psychiatric issues of HD patients, as well as medications used to treat them and possible pharmacokinetic considerations for patients with CKD to aggregate as much data as possible to help clinicians in their daily practice. Furthermore, to search for published deprescribing algorithms used in different populations, as well as CKD patients, that could possibly be implemented in HD patients. Figure 1 summarizes the covered topics of this review.

## Methods

This manuscript represents a narrative review. A literature search was conducted by two authors who identified English manuscripts from Pubmed as well as selected printed works. All types of manuscripts were used, i.e., case reports, observational studies, randomized controlled trials, reviews, or meta-analyses. The authors agreed upon the use of various keywords as well as their possible combinations. The keywords used were: chronic kidney failure; chronic kidney disease; hemodialysis; kidney replacement therapy; polypharmacy; depression; psychosis; dementia; antidementia drugs; donepezil; galantamine, rivastigmine; memantine; antidepressants; escitalopram; sertraline; duloxetine; mirtazapine; antipsychotics; haloperidol; quetiapine; risperidone; aripiprazole; olanzapine; deprescribing algorithms; psychotropic drugs; drug to drug interactions; DDIs; adverse effects; pruritus; and gabapentinoids.

## Psychotropic Drug Interactions

Recently, growing numbers of patients seek support for a wide range of psychiatric illnesses, where some reports show that primary care physicians prescribe 65 and 80% of all anxiolytic and antidepressant drugs, respectively. As patients with psychiatric illnesses can also suffer from several somatic comorbidities, the risk for DDI increases exponentially. These interactions can possibly be pharmacodynamic, i.e., simultaneously administered agents target similar action sites to produce additive or antagonistic effects resulting in enhanced or diminished physiologic response, or pharmacokinetic, i.e., one administered agent effects the absorption, distribution, metabolism, or excretion of another agent resulting in an increase or decrease in the drug’s serum concentration (5). Additionally, prescription errors can occur on average 8.8 times per 100 medication orders. They are classified as omission error (deletion of a drug previously used), commission error (addition of a previously non-used drug), dosing error, frequency error, form error, substitution error (substituting one drug for another from the same class), and duplication error (two drugs from the same class used) (6).

An analysis based on German insurance claims data looked at concomitant prescription of drugs that interact *via* CYP450. They identified 186 (15.2%) patients with at least one drug-drug exposure, which were in 40% identified as clinically relevant. Moreover, 59 (4.8%) patients with medications involving a strong CYP450 inhibitor, where 87.5% were identified as clinically relevant (7). Another German study looked at elderly inpatient hospital cases. They observed that one third (*n* = 15,690) of the patients received at least 5 different medications daily and that 24,006 (51%) received more than one psychotropic drug daily, exposing them to possible DDI and inappropriate medication use (8). Furthermore, an additional factor in possibility of DDI is the increasing use of psychotropic medication, as studies show an 40.1% increase in antidepressant and antipsychotic number of medications between 1996 and 2006, resulting in psychiatric polypharmacy, where many combinations are of unproven efficacy (9). Importantly, as utilization of marijuana and other cannabinoids is increasing Rong et al. have carried out a narrative review of possible DDI (10). They concluded that Δ^9^-tetrahydrocannabinol and cannabidiol are substrates and inhibitors of CYP3A4, CYP2C9, and CYP3A4, CYP2C19, respectively, and advised caution when prescribing psychotropic medications who are similar CYP substrates (10). The impact of HD sessions on changes in metabolism of different medications are not well understood. Egeland et al. have tested the impact of changes in uremic milieu between HD sessions to uncover that each HD session temporarily reduced chronic inhibition of CYP3A but had no impact on P-glycoprotein/organic anionic-transporting polypeptide activity (11).

Elderly patients are at a higher risk for polypharmacy, which is why Das et al. have investigated medications with an impact on the QT interval, which relates to a higher risk of cardiovascular death and all-cause mortality (12). They identified that antidepressant, proton pump inhibitor, antipsychotic, antinausea, antimicrobial, and H2 receptor antagonist medications have highest interactions with inclination to toward QT-interval prolongation. They concluded that reliable evidence-based online drug knowledge resources, such as AzCERT/CredibleMeds Drug Lists, Medscape Drug Interactions Checker, Epocrates Online Interaction Check, and Drugs.com should be adopted to facilitate medication selection (12). A recent retrospective analysis conducted in 10 psychiatric hospitals in Germany has shown that 22,739 (83%) cases received 1–8 QT interval prolonging medications simultaneously, of which pipamperone, quetiapine, prothipendyl, and risperidone were the most common. They concluded that replacement of high-risk drugs such as tricyclic antidepressants, levomepromazine, melperone, and promethazine could avoid 11% of QT prolonging drugs (13).

Similarly, Boyce et al. have studied evidence-based literature and medication package inserts to report age related clearance changes for 13 antidepressants, while they identified 45 medications that could potentially interact with antidepressants to cause a lower clearance or raise in the area under the concentration time curve (14). In a study by Hahn et al., they showed that the introduction of a pharmacist in the psychiatric intensive care unit reduced all drug interactions by 44% (15). Endres et al. calculated that psychiatric patients in German speaking countries had a median of 7 active substances prescribed, which mathematically resulted in 21 interactions (16). Of the most important interactions between antihypertensive and psychotropic medications they reported possibility of hypotension, insufficient blood pressure reduction, or QT interval prolongation (16). Aroke et al. have studied psychotropic therapy prescription after incident cancer diagnosis. Psychotropic polypharmacy was reported in 415 (7.4%) patients with the highest prevalence in lung cancer (14.4%) (17). Additionally, they observed an increase in the use of psychotropic medications after cancer diagnosis and patients with polypharmacy required a higher degree of healthcare services (17). Sun et al. have used a network approach, which included 28 schizophrenia and 241 non-schizophrenia drugs to identify 991 possible drug interactions (18). They reported that typical schizophrenia drugs had the most significant interactions with drugs of the “alimentary tract and metabolism” category, while atypical schizophrenia drugs had significant interactions with drugs of the “nervous system” and “anti-infective for systemic uses” categories. The three typical schizophrenia drugs with most interactions were zuclopenthixol, thiothixene, and thioridazine, and the three atypical schizophrenia drugs with most interactions were ziprasidone, clozapine, and amisulpride, respectively (18).

In regard to the coronavirus disease 2019 (COVID-19) pandemic and several emerging therapies, which could cause significant DDI, Plasencia-García et al. have conducted a database and systemic review (19). Of main concern were QT interval prolongation, Torsade de Pointes, and possibility of CYP450 interaction. They concluded that remdesivir, favipiravir, tocilizumab, baricitinib, and anakinra had little to no evidence about significant interactions with antipsychotics. However, for hydroxychloroquine, chloroquine, azithromycin, and lopinavir/ritonavir several interactions existed, especially when coadministered with chlorpromazine, haloperidol, levomepronacine, ziprasidone, and zuclopenthixol. The safest antipsychotics for coadministration appeared to be asenapine, brexpiprazole, cariprazine, lurasidone, and olanzapine. Nonetheless, caution has to be administered when prescribing COVID-19 therapies and antipsychotics, especially when lopinavir/ritonavir is prescribed (19).

A possible managing tool to avoid clinically relevant DDI is the use of therapeutic drug monitoring (TDM). Spina et al. have published tables which combine available information to help manage average patients with second generation antipsychotics taking inducers or inhibitors of CYP450 and lists possible DDI (20). They identified potent inducers, such as carbamazepine, phenytoin, and phenobarbital, and potent inhibitors such as paroxetine, and recommended possible actions to take (20). Unterecker et al. have used TDM of antidepressants in HD patients (21). They observed that HD patients received rather low doses of antidepressants and that serum concentrations of amitriptyline/nortriptyline and mirtazapine decreased during HD (21). Jacob et al. described a case report of a smoking patient with schizophrenia undergoing HD (22). They found no evidence of HD influence on serum levels of clozapine and concluded that low levels were likely due to CYP1A2 induction by smoking (22). Another case report by Railton et al. using TDM showed that HD did not enhance the elimination of risperidone (23). They concluded that the reason for lower serum levels of risperidone is likely to be reduced absorption due to long periods of peritoneal dialysis the patients received prior to HD and possible peritoneal fibrosis (23).

## Cognitive Impairment

Chronic kidney disease is an independent and significant risk factor for cognitive impairment. The prevalence of cognitive impairment in HD patients is at least two times higher than that of age-matched controls. The pathophysiologic mechanisms postulated for cognitive impairment are vascular injury and direct neuronal toxicity of uremic toxins. Dementia related to vascular causes is more likely to be present than Alzheimer’s disease (AD) in the HD population. The dialysis physician should be alert to warning signs, such as when a patient is demonstrating new behavioral changes, non-compliance with medications and the HD procedure, and is repeatedly asking the same questions (24).

Murray et al. evaluated 374 HD patients finding that only 49 (13%) had normal cognitive function, while 187 (50%) had mild to moderate impairment and 138 (37%) had severe impairment (25). Rates of AD dementia in patients with CKF appear similar to rates in patients without CKF of similar age and a similar burden of comorbid conditions. In contrast, patients with CKF are significantly more likely to have disproportionate levels of cerebrovascular disease, particularly small-vessel cerebrovascular disease. Those with CKF are more likely to experience clinical cerebrovascular disease (incl. stroke and transient ischemic attack), as well as have subclinical cerebrovascular disease on imaging such as small-vessel infarcts, lacunes, and white matter disease (2). What is more, the HD procedure may promote cognitive impairment by sudden hemodynamic shifts, routine use of anticoagulation that may predispose to microbleeds, and intermittent rather than continual solute clearance (2).

Although treatment of CKF with maintenance HD using high-flux high-efficiency membranes and monthly assessment of dialysis adequacy has eliminated much of severe cognitive impairment (encephalopathy) associated with uremia, current dialysis membranes are much less efficient at clearing medium-sized and highly protein-bound metabolites. The 4-hydroxyphenylacetate was recently identified as potentially contributing to CKF-related cognitive impairment (2). Rapid fluid shifts during HD can often lead to wide swings in blood pressure (2). Intradialytic hypotension has been linked with cerebral atrophy, while hemodynamic instability on HD has also been associated with brain injury (2). MacEwen et al. found that nearly a quarter of 635 individual HD sessions showed evidence of cerebral ischemia, with a third of those events being symptomatic (26). The study of Polinder-Bos et al. demonstrated that initiation of HD in elderly individuals resulted on average in a 10% decline in cerebral blood flow, with every brain location/volume showing declines (27). Higher dialysate temperature, ultrafiltration rates, and volume were associated with lower cerebral blood flow (27). Findlay et al. showed that greater decreases in cerebral blood flow were associated with worse cognitive function in addition to progression of white matter disease, as measured by brain magnetic resonance imaging (28). Delivering dialysis to an individual with advanced dementia is challenging and may even be a reason to consider such treatment futile (2).

### Antidementia Drugs

The antidementia drugs Are generally divided Into acetylcholinesterase inhibitors (AchEI) donepezil, galantamine, and rivastigmin and the *N*-methyl-D-aspartate (NMDA) antagonist memantine. Officially the AchEI Were approved for mild to moderate AD, while memantine was approved for moderate to severe AD (29). A possible additional compound Is the Ginkgo biloba extract with its antioxidant properties, however, Its efficacy remains controversial (30). On the other hand, the efficacy of antidementia drugs Is well documented and some studies even show a possible mortality benefit for patients With AD (31–33). Recently, combining both drug classes with medications such as galantamine and memantine has proven to be effective in several neurodegenerative diseases (34). A meta-analysis pooled all adverse effects for AchEI and memantine and reported gastrointestinal adverse effect to be most common, such as nausea, vomiting, diarrhea, and anorexia (33).

#### Donepezil

Donepezil is a second generation AchEI. Possible routes of administration include only peroral tablets, however, liquid and transdermal formulations have also been developed. Generally, it is dosed 5 mg daily, with an increase to 10 mg after 1 month (35, 36). Recent animal studies also show the possibility of intranasal application (37). Its volume of distribution is 12 L/kg, it is 95% protein bound, and its half-life lasts 70 h. It is metabolized in the liver by the cytochrome (CYP)3A4 and CYP2D6 and is excreted (17% unchanged) in the urine in 57% and feces in 14.5% (35). A Cochrane review showed several possible adverse effects, which were mild and rare. Patients receiving donepezil had more anorexia, diarrhea, dizziness, fatigue, hallucinations, insomnia, muscle cramps, nausea, peripheral edema, tremor, vertigo, vomiting, and weight loss compared to placebo (36). A comparative study by Fleet et al. reported a slightly higher, yet still very low, 30-day risk of admission to hospital with rhabdomyolysis compared to rivastigmine or galantamine (38).

Amano et al. have studied the use of donepezil in patients with CKF. They concluded that cognitive function was improved best when the 5 mg daily dose was used, however, the plasma level came close to reaching a toxic level (39). Yiannopoulou et al., have reported a case series of five patients on maintenance HD with AD prescribed donepezil (40). They started with a dose of 2,5 mg daily and increased it after 1 month to 5 mg daily. No adverse effects were observed. They observed a slight improvement in cognitive and executive functions. During 10 years of follow up, the patients showed a mild cognitive decline per year for the first 5 years, which progressed to severe cognitive decline for the remaining years of follow up (40).

#### Galantamine

Galantamine is a second generation AchEI. It is administered perorally in regular and extended-release formulations and dosed between 4 and 12 mg twice daily for the regular formulation and 8–24 mg for the extended release. Its bioavailability is around 70%, volume of distribution between 175 L/kg, it is 18% protein bound, and its half-life ranges between 7 and 10 h. It is metabolized in the liver *via* CYP2D6 and CYP3A4 up to 75%, where active metabolites are formed. It is excreted (20–30% unchanged) in the urine and up to 6% in the feces (35). The exposure to galantamine in severe renal impairment is 67% higher than in healthy subjects and it is likely to be dialyzed (35, 41). To the best of our knowledge, we have not found any published work regarding use of galantamine in CKD or HD.

#### Rivastigmine

Rivastigmine is a second generation AchEI and the only AchEI that also inhibits butyrylcholinesterase in the brain. It is available in peroral formulation, which is administered at doses of 1.5 mg twice daily with an increase to 3–6 mg twice daily, and in transdermal formulation dosed 4.6–13.3 mg/24 h patch daily. Its volume of distribution ranges between 1.8 and 2.7 L/kg, it is 40% protein bound, and its half-life is 1 h. It is metabolized *via* the cholinesterase-mediated hydrolysis and excreted in the urine in >90%. It is likely to be dialyzed (35, 42). To the best of our knowledge, we have not found any published work regarding use of rivastigmine in CKD or HD.

#### Memantine

Memantine is a NMDA receptor antagonist. It can be administered perorally in doses between 5 and 20 mg daily. Its volume of distribution is 10 L/kg, it is 45% protein bound, and its half-life ranges between 60 and 100 h. It is partially metabolized in the liver and is excreted (48% unchanged) in the urine with active tubular secretion. It is likely to be dialyzed (35). Pharmacokinetic studies show that mild, moderate, and severe kidney impairment results in 1.62-, 1.97-, and 2.33-times higher plasma concentration-time curves, respectively, which is why the dose must be adjusted (43). It is recommended to use a target dose of 10 mg daily, which is half of the recommended dose, when the creatinine clearance (CrCl) < 30 ml/min (44).

Dolder et al. have performed a retrospective analysis to report only 28 (40%) patients had a kidney adjusted dose of memantine prescribed (45). Pei et al. have reported a case of a patient with non-adjusted dose of memantine who developed new onset myoclonus, which resolved 2 days after cessation of memantine (44). Additionally, Hurikawa et al. have reported a case memantine associated subacute kidney injury, which developed 14 days after initiation of treatment and returned to normal after cessation of memantine (46).

## Depression

Depression in HD patients may be a result of poor dialysis tolerance, inadequate pain control, and other unfavorable social situations (24). The prevalence of major depression in stage 5 CKD varies between 14 and 30% (47). Considering HD patients, a higher frequency of depression was observed in DM patients (42.9% compared to 24.3% in non-DM patients). Additionally, patients who used more than five medications had a higher prevalence of depression (38.7% compared to 24.5%) (29). Depressed patients had significantly lower average albumin and creatinine levels than non-depressed patients. In the study of Teles et al. nearly one-third of the sample (*n* = 200) reported symptoms compatible with depression, but only 8 (4%) patients used antidepressants (48). Only a minority of depressed patients on HD receive adequate drug treatment for depression (47, 48). It has been demonstrated that providing pharmacologic treatment to depressed patients on HD improves their nutritional parameters and reduces their inflammatory activity (48). However, there is no high-quality evidence from randomized trials that suggests antidepressants are more effective than placebo in treating depression in patients with CKD stage 3–5. The available data point toward needing a different time for improvement after antidepressant therapy initiation for patients with CKD (up to 12 weeks) in comparison with the general population (47).

### Antidepressants

Antidepressants are divided into so-called older and newer antidepressants. The group of older antidepressants involves heterocyclic antidepressants that include tricyclic tertiary amines and their secondary amine metabolites (TCAs), such as imipramine, amitriptyline with its metabolites desipramine or nortriptyline, and modified tricyclic antidepressants (tianeptine). Monoamine oxidase inhibitors (MAOIs) are still sometimes used for the treatment of major depressive episodes. Older antidepressants, such as TCAs, are associated with numerous side effects as a result of cholinergic, histaminergic, and alpha-adrenergic receptor antagonism. Newer antidepressants include selective serotonin reuptake inhibitors (SSRIs), such as citalopram, escitalopram, fluvoxamine, fluoxetine, paroxetine, and sertraline; serotonin modulators and stimulators (SMSs), like vortioxetine, or vilazodone; serotonin antagonist and reuptake inhibitors (SARIs), e.g., trazodone and nefazodone; SNRIs, e.g., venlafaxine, duloxetine, or milnacipran; norepinephrine-dopamine reuptake inhibitor (NDRIs), e.g., bupropion; norepinephrine reuptake inhibitors (NARIs); and noradrenergic and specific serotonergic antidepressants (NaSSAs), e.g., mirtazapine, or mianserin (49).

Most of the newer antidepressants share several common features, such as good absorption from the gastrointestinal tract into blood, a highly variable bioavailability, and extensive distribution into tissue. They are extensively metabolized in the liver by cytochrome P450 isoenzymes. As the main mechanism of elimination is by the liver, they do not accumulate significantly even in severe renal impairment. However, their metabolites are renally excreted and may accumulate in patients with decreased glomerular filtration rate (GFR) (49). Newer antidepressants are generally highly protein bound, thus it may be expected that they will not be removed significantly by HD (49).

Although few controlled studies have evaluated the safety of antidepressant medication in HD patients, serotonin reuptake inhibitors are considered safe drugs (48). Most of the studies included in the review of Constantino and Fonseca found no differences in the pharmacokinetics of antidepressant drugs between patients with normal renal function and patients undergoing HD. However, studies with fluvoxamine and amitriptyline showed that variations in albumin levels might affect serum levels of these agents (50). A systematic review of randomized clinical trials and observational studies examining antidepressants in patients with CKD stage 3–5 concluded that dose reduction in CKD stage 3–5 is necessary for amitriptylinoxide, venlafaxinemilnacipran, and bupropion (47).

### Selective Serotonin Reuptake Inhibitors

Selective serotonin reuptake inhibitors (SSRIs) are the first-line treatment in major depression due to their improved tolerability and safety profile in relation to the conventional antidepressants. Maximal antidepressant effects are observed after 2–3 weeks of chronic treatment. Currently used SSRIs (citalopram, escitalopram, fluvoxamine, fluoxetine, paroxetine, and sertraline) have similar antidepressant efficacy and safety profiles, but they differ in their chemical structure, receptor affinity, and psychochemical and pharmacokinetic properties (49). Fluoxetine can accumulate extensively following multiple oral administrations and steady state concentrations are usually achieved after a long time; 3–4 weeks after initiation of therapy (49).

#### Escitalopram

The usual daily dose is 10–20 mg. Its volume of distribution is around 12–26 L/kg, it is less than 80% protein bound, and its half-life is about 22–32 h, slightly increased in CKF. It undergoes hepatic metabolism mainly by CYP2C19 and is generally excreted in the urine (8% unchanged). The recommendation about dosing in estimated GFR < 30 ml/min and in HD patients is to start with a low dose and titrate slowly (35).

#### Sertraline

The usual daily dose is 25–200 mg. Its volume of distribution is 25 L/kg, it is >98% protein bound, and its half-life is 26 h. It undergoes extensive first-pass metabolism in the liver and is excreted equally through the feces and urine. It is not dialyzed. The dosage is the same as in normal renal function (35). According to the studies of Schwenk et al. post-HD supplementation is unnecessary since absorption and distribution of the drug is not altered by HD (51).

### Serotonin-Norepinephrine Reuptake Inhibitors

Antidepressants from this group have a dual mode of action: they inhibit the reuptake of serotonin and noradrenaline (NA) with different selectivity. In this group of antidepressants, the steady state is achieved much faster (4–5 days) than in the case of SSRIs. They have different pharmacokinetic properties in comparison to SSRIs, mainly because of much shorter half-life values and lower protein binding. The only exception to this is the most frequently used serotonin-norepinephrine reuptake inhibitors (SNRI), duloxetine (49).

#### Duloxetine

The normal daily dose is 60 mg. It is around 95% protein bound. It is extensively metabolized and less than 1% is excreted unchanged in the urine, while 77% is excreted as metabolites. Its volume of distribution is around 1,640 L/kg. The half-life is around 8–17 h and is unchanged in CKF. It is contraindicated by manufacturer if creatinine clearance (CrCl) falls below 30 ml/min. It is not dialyzed (35).

### Other Newer Antidepressants

Mirtazapine is currently the only used NaSSA. It is rapidly absorbed from the gastrointestinal tract, although its bioavailability is not very high because of gut wall and hepatic first-pass metabolism. The peak plasma concentrations are reached within 2 h (49). The usual daily dose is 15–45 mg once or twice daily. The volume of distribution is 107 L/kg, it is around 85–90% protein bound, 75% is excreted unchanged in the urine, its half-life is 20–40 h, which is increased with renal insufficiency. It is unlikely to be dialyzed. If GFR is less than 20 ml/min low dose and close monitoring are indicated (35).

The results of the older studies of Unterecker et al. and Bengtsson et al. suggested potential accumulation in patients with renal dysfunction and showed a significant decrease of mirtazapine concentration after HD (21, 52). A newer study of Schlotterbeck et al. showed that low-flux membrane, did not show any significant effect on plasma concentrations of mirtazapine, however, nowadays the low flux membranes are rarely used (53).

## Antipsychotics

Antipsychotics are an important part of the psychiatric management, that were established as the cornerstone of treatment in schizophrenia several decades ago. Their development went through several phases. The first antipsychotics developed in the 1950s have shown to be groundbreaking in the field of schizophrenia and have led to deeper understanding of the importance of dopamine blockade in psychiatric illnesses (54). As our experience with their use grew, several adverse effects were established, such as cardiovascular effects and hyperprolactinemia, with extrapyramidal (motor) symptoms playing an important role in the pursuit of alternative medications. Second generation (atypical) antipsychotics were developed with a better safety profile, however, displayed several cardiovascular and metabolic adverse effects. Further development due to adverse effects has established a third generation of antipsychotics, which appear to have a better metabolic safety profile and appear to specifically target negative symptoms and cognitive domains (55). This has led to a shift toward prescription of atypical antipsychotics, with 93% of all antipsychotic agents prescribed in the United States of America (USA) in 2008 representing atypical antipsychotics (56). Additionally, Buhagiar et al. reported that they observed a 19.2% rise in antipsychotic prescription rate in United Kingdom between 2011 and 2016 (57). Similarly, Lao et al. have reported an increase in prevalence of antipsychotic prescribing from 1.06 to 1.54% in the general population in Hong Kong (58).

Antipsychotics were initially approved for the treatment of schizophrenia, but their use has nowadays expanded. They are prescribed, sometimes off-label, for psychoses, bipolar disorder, delirium, depression, personality disorders, dementia, and autism (56). Lao et al. reported that more than 50% of incident users in 2014 had non-psychotic mental illnesses (58). In 2008 the most common typical agent prescribed in the United States was haloperidol and the most prescribed atypical agents were quetiapine, risperidone, aripiprazole, and olanzapine (56).

### Haloperidol

Haloperidol is a first generation (typical) antipsychotic drug. It acts as an antagonist of dopamine 1 (D1) and 2 (D2) receptors, while also exhibiting some affinity toward serotonin [5-hydroxytryptamine (5-HT)] and histamine (H1) receptors, and α1 adrenoreceptors (59). Possible routes of administration include peroral, intramuscular, subcutaneous, and slow bolus intravenous. Generally, it is dosed between 1 and 10 mg depending on the indication with the possibility of repeated dosing. Additionally, it is possible to administer a deep intramuscular dose of up to 300 mg monthly (35).

Its bioavailability ranges between 60 and 70%, volume of distribution between 14 and 21 L/kg, it is 90–92% protein bound, and its half-life ranges between 15 and 37 h (36, 60, 61). It is metabolized in the liver *via* CYP3A4 and CYP2D6, and excreted (1% unchanged) in the urine and bile (35). Due to its protein binding and volume of distribution it is unlikely to be removed by HD. Some variations in plasma levels could be due to changes in volume status and hypoalbuminemia that occur during HD sessions, where some reports have noticed a need for slightly higher dosing in HD, although it is usually recommended to start with lower doses in patients with an estimated GFR < 10 ml/min (35, 61, 62). The most predominant among its adverse effects are extrapyramidal symptoms (dystonia, parkinsonian-like syndrome, and tardive dyskinesia). Other adverse effects include anticholinergic effects (constipation, dry mouth, blurred vision, and urinary hesitancy), sexual dysfunction, hyperprolactinemia, QT interval prolongation, and sedation (59, 60). Importantly, patients receiving HD treatments are usually anticoagulated, which is why intramuscular injections should generally be avoided due to possible hematoma risk.

### Quetiapine

Quetiapine is a second generation (atypical) antipsychotic drug. It acts as a D2 receptor antagonist, that rapidly dissociates from the receptor. Furthermore, it has a pronounced affinity for the α1 adrenoreceptors and H1 receptors, while also exhibiting some affinity for the muscarinic acetylcholine (mACh) and 5-HT receptors (59, 63, 64). It is administered perorally in regular and extended release formulations and dosed between 50 and 750 mg once or twice daily depending on the indication (35).

Its bioavailability is around 70%, volume of distribution between 6 and 14 L/kg, it is 83% protein bound, and its half-life ranges between 5 and 7 h (35, 61). It is metabolized in the liver *via* CYP3A4, and excreted in 73% (<5% unchanged) in the urine and in 21% in the feces as inactive metabolites (35). The available pharmacokinetic studies have shown no important difference in patients with CKD, however, no studies have been carried out regarding pharmacokinetics of quetiapine in HD (61). A small cross-sectional study from India reported the use of quetiapine in some HD patients, however, did not mention adverse effects and reported use of lower doses (65). As a second-generation antipsychotic it does not show extrapyramidal symptoms, however, reported adverse effects include hypotension, midrange QT interval prolongation, modest weight gain, and some gastrointestinal and anticholinergic effects. Additionally, some cases have reported neutropenia and agranulocytosis (63).

### Risperidone

Risperidone is a second generation (atypical) antipsychotic drug. It acts as a serotonin dopamine antagonist, blocking D2 and 5-HT receptors, with a 5-HT_2*A*_/D2 affinity ratio of about 20, while also exhibiting affinity for the α1 and α2 adrenoreceptors and H1 receptors (59, 63, 64). It is available in peroral formulation, which is administered at doses of 0.25–16 mg daily depending on the indication, however, can also be administered as long acting injectable (LAI) risperidone microspheres intramuscularly in doses of 25–50 mg every 2 weeks (35). Intramuscular application during HD is usually not advised due to frequent use of anticoagulation.

Its bioavailability ranges between 70 and 85%, volume of distribution between 1 and 2 L/kg, it is 90% protein bound, and its half-life ranges between 3 and 30 h (35, 63). It is metabolized in the liver *via* CYP2D6 to its active metabolite paliperidone, and excreted (70% unchanged) in the urine and to a lesser extent in the feces (35). Based on observational studies, case reports and known high protein binding of risperidone it is unlikely to be dialyzed and therapeutic drug levels seem to not be affected by HD, except due to hypoalbuminemia and changes in volume status. This goes for regular and LAI risperidone. Additionally, researchers observed higher plasma concentrations in patients on HD, which could be due to accumulation in CKD (61, 66). On the other hand, some report that about 25% of risperidone is removed after a 5-h dialysis session with a dialysate flow of 500 ml/min. All this resulted in recommendations for dosing in CKD, which should be initiated and titrated at 50% the usual dose once the estimated GFR < 50 ml/min (35, 61). Important adverse effects include low-to-modest weight gain, increase in cerebrovascular events in the demented elderly, low range QT interval prolongation, hypotension. Additionally, some cases have reported neutropenia and agranulocytosis (63).

### Aripiprazole

Aripiprazole is a third generation (atypical) antipsychotic with partial agonistic effects on D2 and 5-HT_1*A*_ receptors and antagonistic activity at 5-HT_2*A*_ receptors (59, 63, 64). It is available in regular and LAI formulations. Regular can be administered perorally in doses between 10 and 30 mg daily or intramuscularly in doses between 5.25 and 15 mg daily, which can be given three times daily. The LAI formulation is given intramuscularly 400 mg monthly (35). As mentioned previously, intramuscular injection is not advised during HD treatment.

Its bioavailability is 87%, volume of distribution is 4.9 L/kg, it is >99% protein bound, and its half-life ranges between 47 and 146 h (35, 63). It is metabolized in the liver *via* CYP3A4 and CYP2D6 to its active metabolite dehydro-aripiprazole and is excreted in 25–27% (<1% unchanged) in the urine and in 55–60% (18% unchanged) in the feces (35, 64). According to current recommendations on dosing and manufacturer’s instructions no dosing adjustments are required regarding age, sex, kidney, or liver function (16, 46). This was also confirmed in a case report when using LAI aripiprazole in HD (67). However, one case report noticed worsening behavior on days after dialysis when aripiprazole was given prior to HD, which is why they suggested dosing after HD and around the same time on non-dialysis days (68). As a third-generation antipsychotic it appears to evade several known adverse effects of antipsychotics. It has a low-grade effect on QT prolongation and an expert review concluded that it is a generally well tolerated drug with low rates of motor and metabolic adverse effects (63, 69).

### Olanzapine

Olanzapine is a second generation (atypical) antipsychotic with high affinity for D2 and 5-HT_2*A*_ receptors, with some affinity for D1 and muscarinic (M1) receptors (59, 64). It is available in regular and LAI formulations. Possible routes of administration are peroral, where it is dosed 5–20 mg daily, and intramuscular, where it is dosed 5–10 mg for up to three times daily. The LAI formulation can be administered in a dose of 150–300 mg every 2 weeks or 300–405 mg every 4 weeks (35). Intramuscular injection is not recommended during HD sessions.

Its bioavailability is 60%, volume of distribution is 10–20 L/kg, it is 93% protein bound, and its half-life ranges between 30 and 38 h (35, 63). It is metabolized in the liver *via* CYP1A2 and, to a lesser extent, CYP2D6 to inactive metabolites and is excreted (7% unchanged) in 57% in the urine and in 30% in the feces (16, 46). Dosing in CKD must be adjusted and current recommendations suggest dosing 5 mg daily or 150 mg for LAI, which must be titrated as necessary (35). In patients on HD some case reports have warned against use of LAI as it can cause significant sedation and monitoring could be a challenge during HD (61). A case report has also mentioned possibility of hypothermia in a patient on HD receiving olanzapine for night delirium (70). Olanzapine, next to clozapine, is associated with the greatest risk of weight gain. Other adverse effects include an increase in cerebrovascular events in the demented elderly, treatment induced diabetes mellitus, dyslipidemia, low-range QT interval prolongation, hypotension, neutropenia, and agranulocytosis (63).

## Discussion

Elderly patients are prescribed a median of 7 medications, while patients undergoing HD receive on average 10–17 medications, as they often have comorbidities such as DM, AH, or CVD, with 70% of these medications being potentially inappropriate. An additional difficulty is the fact that a typical patient on dialysis has 4 different prescribers (71–73). This can result in medication related problems. A study by Alshamrani et al. on HD patients has identified several medication related problems, such as medication use without indication in 30 (36%) patients, subtherapeutic dosing in 19 (23%) patients, and overdosing in 12 (15%) patients (3).

Avoiding or minimizing polypharmacy, be it for somatic or psychiatric illnesses, can be difficult but may be achieved in utilizing different algorithms for medication use. With ageing, patients’ goals of treatment may change due to their physical and functional condition alterations. A possible method to tackle this is called deprescribing, which is defined as a patient centered process of stopping or reducing the dose of medications that are inappropriate for the patients as their risks outweigh potential benefits or they are ineffective (74). One of the best implicit tools, The Medication Appropriateness Index (MAI), was published in 1992 and is used to assist in recognizing prescribing errors and improving overall prescribing quality in older people. It is comprised of 10 questions used to identify a variety of potential prescribing errors (6, 75).

Recently, an explicit tool has emerged and showed clinical benefit in several trials. The Screening Tool of Older Persons Prescriptions and Screening Tool to Alert doctors to Right Treatment (STOPP/START) criteria were first published in 2008 and received a second iteration in 2015. The first version lists 87 criteria, while the second version was expanded to include 114 criteria. START criteria are focused on possible prescription omissions, while STOPP criteria help to identify common and potentially inappropriate medications (6, 76–78). Five single center trials showed, that using these criteria improves medication appropriateness, reduces cost, falls, and adverse drug reactions (ADRs) (78). Recently two large randomized controlled trials were published examining the use of STOPP/START criteria, namely Optimizing Therapy to Prevent Avoidable Hospital Admissions in Multimorbid Older Adults (OPERAM) and Software ENgine for the Assessment and optimisation of drug and non-drug Therapy in Older peRsons (SENATOR). OPERAM showed that 789 (86.1%) participants in the intervention arm had inappropriate prescribing resulting in 2.75 recommendations per participant. However, the interventions had no effect on drug related hospital admissions (79). Similarly, the SENATOR trial showed no difference in ADRs, however, implementation of software-generated advice was low at 15% (80).

Two additional tools that have gained recognition especially in the elderly are American Geriatrics Society’s Beers Criteria that were updated in 2019 and the FORTA (Fit-fOR-The-Aged) list (81, 82). The Beers criteria list tables with medications that are potentially inappropriate for most elderly patients, for those with certain health conditions, and those which should be used with caution, as well as listing those which could have important DDIs or should be adjusted according to kidney function (82). Grina and Briedis showed with a retrospective, observational study including 431,625 patients, that 25.9% had potentially inappropriate medications based on Beers 2015 criteria, of which benzodiazepines were the most common (83). Another study by Gorzoni and Rosa in 39 patients aged >80 years showed that each patients has 1.8 potentially inappropriate medications prescribed according to Beers criteria, mostly from the “if necessary” group (84).

Furthermore, European experts have published in 2015 The EU(7)-PIM list, which identified 282 chemical substances or drug classes from 34 therapeutic groups, which represent potentially inappropriate medications for the elderly and provided suggestions for dose adjustments and therapeutic alternatives (85). Williams et al. have used the above-mentioned tools to identify that anticholinergics, benzodiazepines, antipsychotics, and opioids are potential medication classes that have been shown to be successfully deprescribable in the elderly (71). Triantafylidis et al. have highlighted specific targets for optimization or deprescription in older patients with CKD that included proton pump inhibitors, oral hypoglycemic agents, and statins (86).

The process of deprescribing consists of several steps. It begins with comprehensive medication reconciliation, followed by identification of essential and potentially inappropriate medication, and ends with discussing potential benefits and risks of deprescribing with the patients and reaching an agreement (74). A systemic review and meta-analysis reported that medication deprescribing interventions may provide small reductions in mortality and use of potentially inappropriate medications in community dwelling people aged above 65 years (87). George et al. have published a study proving that deprescribing for HD patients is feasible and safe (88). They observed a significant reduction in the number of medications from 11 to 8 and reduced the pill burden from 16 to 11 (88). A study done by Bondurant-David et al. showed that HD patients view deprescribing as favorable because it presents them with an opportunity to discuss their ambivalence toward medication and empowers them as a patient partner in their care (72).

Reeve et al. have published a guideline for successful deprescribing cholinesterase inhibitors and memantine in dementia (89). Individuals who may be suitable for a trial of deprescribing are those without an appropriate indication, without benefit, or with severe or end-stage dementia. They concluded that this approach may improve quality of life with reduced medication burden and adverse effects (89). Pottie et al. have published a clinical practice guideline to deprescribe benzodiazepine receptor agonists for patients using them for primary insomnia or comorbid insomnia when the underlying conditions are effectively managed (90). They suggested a slow taper for patients who are above 65 years or between 18 and 64 with use of a benzodiazepine receptor agonist for more than 4 weeks (90). Bjerre et al. have recommended a clinical practice guideline for deprescribing antipsychotics in adults with behavioral and psychological symptoms of dementia (91). Deprescribing should be attempted in patients who were treated for 3 months and achieved symptom stabilization or no response. Furthermore, this should be tried for patients with primary insomnia or secondary insomnia with managed underlying comorbidities (91). Lefebvre et al. have developed and validated nine deprescribing algorithms for HD patients. They published recommendations for deprescribing alpha-1 blockers, loop diuretics, proton pump inhibitors, quinine, statins, benzodiazepines and Z-medications, gabapentinoids, prokinetic agents, and urate-lowering agents (92). To the best of our knowledge, we have not found any deprescribing algorithms for antipsychotics or antidementia drugs specifically in HD patients, possibly due to lower rates of prescribing.

## Conclusion

The HD patient population is a large, heterogenous group of people, who is growing older due to numerous advancements in KRT achieved in the last decades. This results in rising incidence of several mental health issues that might need management with psychotropic medication. Introduction of specific therapy is in the domain of psychiatrists and neurologists. However, dialysis physicians and clinical pharmacologists should be consulted for better patient management. As polypharmacy is evidently present in the HD population, the use of deprescribing is highly important.

## Author Contributions

MK and TP: conceptualization and writing—original draft preparation. MK, TP, NP, RE, and SB: writing—review and editing. All authors have read, and agreed to the published version of the manuscript.

## Conflict of Interest

The authors declare that the research was conducted in the absence of any commercial or financial relationships that could be construed as a potential conflict of interest.

## Publisher’s Note

All claims expressed in this article are solely those of the authors and do not necessarily represent those of their affiliated organizations, or those of the publisher, the editors and the reviewers. Any product that may be evaluated in this article, or claim that may be made by its manufacturer, is not guaranteed or endorsed by the publisher.
